# Considerable Genetic Diversity of *Trichomonas vaginalis* Clinical Isolates in a Targeted Population in South of Iran

**Published:** 2017

**Authors:** Razieh TAVAKOLI OLIAEE, Zahra BABAEI, Gholam Reza HATAM, Amir TAVAKOLI KARESHK, Hosein MAHMOUDVAND, Arghavan VAFAFAR, Naser ZIAALI

**Affiliations:** 1. Dept. of Parasitology and Mycology, Kerman University of Medical Sciences, Kerman, Iran; 2. Diagnostic Laboratory Sciences and Technology Research Center, Shiraz University of Medical Sciences, Shiraz, Iran; 3. Dept. of Medical Parasitology and Mycology, Lorestan University of Medical Sciences Khorramabad, Iran; 4. Dept. of Parasitology and Mycology, Shiraz University of Medical Sciences, Shiraz, Iran; 5. Research Center for Tropical and Infectious Diseases, Kerman University of Medical Sciences, Kerman, Iran

**Keywords:** Trichomoniasis, PCR-RFLP, Actin gene, Genotypes, Iran

## Abstract

**Background:**

The present study aimed to characterize genetically and to compare the most frequently occurring strains of *Trichomonas vaginalis* isolated from southern Iran.

**Methods:**

Totally, 150 vaginal swab and urine specimens were collected from symptomatic and asymptomatic women from May 2012 to Jun 2013. This study implemented a sensitive and reliable PCR-restriction fragment length polymorphism (RFLP) typing method on the actin gene. Moreover, one representative sample of each identified genotype was subjected to sequencing.

**Results:**

Twenty-four *T. vaginalis* isolates were positive and 6 distinct electrophoretic patterns (H, E, G, I, M, N) were identified. Genotypes H and I were found to be more prevalent (50 and 37.5%) in Kerman and Shiraz, respectively. The phylogenetic analysis showed that two isolates were located as a separated clade with the other *T. vaginalis* isolates.

**Conclusion:**

The obtained findings showed a considerable genetic polymorphism of clinical isolates from the population studied. More studies may be warranted in future as to unveiling any possible links between a given genotype/cluster and pathogenic behavior of *T. vaginalis.*

## Introduction

Trichomoniasis is the most frequent cause of non-viral sexually transmitted infections in the world and is responsible for more than 180 million new cases each year worldwide (1). This sexually transmitted disease is caused by the anaerobic flagellated protozoan, *Trichomonas vaginalis*. Clinical manifestations in both females and males may vary greatly. For example, in females the disease may range from asymptomatic infections to severe symptomatic cases associated with vaginitis and urethritis, predisposing the patients at the risk of adverse pregnancy outcome and cervical cancer (2, 3). However, clinical symptoms ranging from urethritis and dysuria to chronic prostatitis and infertility may be observed in men (4). Most cases of males and females trichomoniasis are predominantly asymptomatic with any overt clinical manifestations and asymptomatic male patients play a key role in the maintenance of the transmission cycle of the parasite (5).

*T. vaginalis* has a large genome of 160 Mb with approximately 60000 protein-coding genes. Most of its genome contains repeats and transposable elements as their movement through the genome may explain noticeable genetic variability (6).

Actin is one of the structural proteins present in the eukaryotic cytoskeleton encoded by a gene family of at least nine members in *T. vaginalis*(7). This structural protein has a well-conserved ubiquitous nature (8), making it a feasible option for intra-species molecular identification.

There is a little information about the nature and existence of genetic diversity and sequencing analysis of different *T. vaginalis* strains. Random amplified polymorphic DNA (RAPD) technique was used to reveal any possible association between a given genotype and metronidazole resistance in *T. vaginalis* isolates (9). Furthermore, restriction fragment length polymorphism (RFLP) and PCR-RFLP methods were also implemented to assess the level of genetic diversity in clinical isolates of the parasite by different groups (10, 11).

PCR-RFLP is considered by most groups to be an optimal method for strain typing assessment as this method combines the sensitivity of PCR and reliability of RFLP (12). One of the advantages of this method is to allow the identification of the nucleotide substitutions gene tracking of polymorphisms present within the population (11).

There are few investigations on the molecular epidemiology and genetic diversity of *T. vaginalis* strains in Iran (13–15). To achieve more information about genetic properties of *T. vaginalis* isolates from southern Iran, this study was aimed to evaluate actin gene, as a genetic marker, for molecular investigation of the parasite by using PCR-RFLP and DNA sequencing methods.

## Materials and Methods

### Study population

This descriptive cross-sectional study was performed from May 2012 to June 2013. Totally, 150 women in childbearing age with or without clinical symptoms were selected. These women were convicted prisoners from Central Prison of Kerman as well as drug-addicted and HIV-positive women in the same age group of Behavioral health clinic in Shiraz. Patients who referred with symptoms or any history of vaginal discharge and vaginal itching, dysuria and dyspareunia were considered as symptomatic group.

Written consent was obtained from all the patients participating in this study (Project No. 90/258).

### Collection of samples

Vaginal discharge of the posterior fornix was collected by two sterile cotton swabs and the collected samples were immediately transferred into sterile tubes containing 3 mL normal saline as transport medium. For urine specimen, the first-voided urine sample was also collected in a sterile container. All samples were transferred immediately to our laboratory for microscopic examination and subsequent culture.

### T. vaginalis cultivation

Urine samples were centrifuged at 750 g for 5 min and the pellet was immediately applied to glass slide for microscopic wet-mount examination. For the primary isolation, both, the urine pellets and vaginal discharges were inoculated in liquid phase of xenic Dorset culture medium (16, 17) and incubated at 37 °C for up to 5 d. Axenization was achieved by inoculating the isolates into TYI-S-33 medium supplemented with 10% heat-inactivated calf serum, mix vitamin(No.18), streptomycin(100 *μ*g/mL; Sigma) and penicillin(100 U/mL; Sigma) at 35.5 °C for 48 has described before (18). A sample was considered positive for *T. vaginalis* if the microscopy or culture test was positive. Parasites were washed with 7 mL sterile Phosphate Buffered Saline (PBS, pH 7.4) and centrifuged at 3000 g for 7 min for three times. After final wash, the supernatant of each tube was decanted and the pellet was preserved at −20 °C for further analysis.

### DNA extraction

Genomic DNA was extracted from parasites cultured in TYI-S-33 medium using the QI-Aamp DNA minikit (Qiagen, Germany) according to the manufacturer’s instructions. The genomic DNA was eluted in 100 *μ*L of TE buffer (pH 8) and then used as template for PCR.

### Nested PCR and RFLP

The actin gene was selected as a target for PCR amplification by two pairs of primers (11). A pair of external primers including Tv8S (5′-TCTGGAATGGCTGAAGAAGACG-3′) and Tv9R (5′-CAGGGTACATCGTATTGGTC-3′) and the other pair of internal primers Tv10S (5′-CAGACACTCGTTATCG-3′) and Tv11R (5′-CGGTGAACGATGGATG-3′) were used. The PCR master mixture consisted of 2.5 *μ*L 10x PCR buffer, 2.5 mMol/L MgCl2, 200 *μ*Mol/L deoxyribonucleotide triphosphate (dNTP, Fermentas, Thermo Scientific, USA), 25 pMol of each primer (Faza Biotech, Tehran, Iran) (Tv8S, Tv9R) and (Tv10S, Tv11R), 2.5 U/*μ*LTaqDNA polymerase (Fermentas, Thermo Scientific, USA), 2 *μ*L DNA template and distilled water to adjust the final volume to 25 *μ*L.

The same PCR master mixture was used for amplification with the internal primers, and 1 *μ*L of the primary amplified product was added as template. The negative control (PCR master mix with no DNA template) was amplified in parallel with clinical samples. A *T. vaginalis* positive sample confirmed by PCR was considered as a positive control.

PCR amplification was performed in two stages in a FlexCycler (Analytik Jena, Germany). The amplification started with an initial denaturation at 95 °C for 5 min, followed by 10 cycles of 30 sec at 94 °C, 30 sec at 55 °C, and 3 min at 72 °C and a final extension of 7 min at 72 °C. The second stage continued by 25 cycles repetition of the same denaturation and annealing steps with respect to extension step prolonged by 5 sec per cycle. Similar running settings used for the second stage of the nested PCR with the internal primers with a difference in annealing temperature that increased to 61.3 °C.

Five *μ*L of PCR product was analyzed by electrophoresis on 1% agarose gel in Tris-acetate-EDTA buffer (TAE, pH 8), and visualized under UV light by ethidium bromide staining (0.5 *μ*g/mL; Applichem, Biochemica, Germany). The size of the target was 1100 bp, which is only 28 bp shorter than the full length of the open reading frame of the actin gene.

For RFLP, 10 *μ*L amplified product was digested with 0.5 *μ*L of each restriction endonucleases *HindII*(10 U/*μ*L, 500 units), *RsaI*(10 U/*μ*L, 1000 units) and *MseI*(10 U/*μ*L, 300 units), respectively (Fermentas, Thermo Scientific, USA). The reaction was incubated for 4 h at 37 °C in a water bath. The fragments were separated on a 3% agarose gel in TAE buffer as described earlier. Finally, the gel was stained with ethidium bromide and visualized on a translluminator. The size of the amplified products was assessed using a 100-bp commercial weight marker (GeneRuler 100bp DNA Ladder, Thermo Scientific, fermentas).

### Alignment and Phylogenetic analysis

The consensus sequences of the six sequence types (H, M, E, G, I and N), along with the reference sequences obtained from basic local alignment search tool (BLAST) queries were aligned using version 7.0 of the Clustal-W program within the BioEdit and Sequence Scanner V1.0 software package. Sequencing was carried out by Sinaclon CO, using a Source Bioscience Sequencing (Cambridge United Kingdom).

The phylogenetic relationships among genotypes were estimated using Maximum-likelihood analysis. Mega 6 software was also used to construct the phylogeny trees of genotypes. *Tetratrichomonas gallinarum* (GenBank accession numbers: AB468096) was employed as outgroup to root the resulting trees.

### Statistical analyses

Statistical analysis was carried out using SPSS statistical software ver. 18 (Chicago, IL, USA).

Fisher’s exact test was used to determine the relationship between genotypes and presence of clinical symptoms in samples. A *P*-value of less than 0.05 was considered as statistically significant. Moreover, to compare between presence of clinical symptoms and positive culture, Logistic regression was used.

## Results

### Clinical characteristics and Nested PCR

Totally 150 vaginal (n=135, 90%) and urine (n=15, 10%) specimens were isolated from patients in two main cities of Iran, Kerman, and Shiraz. Among participants, 42% (n=63) were found to have some clinical symptoms of trichomoniasis, whereas the remaining 58% (n=87) were asymptotic (Table 1).

**Table 1:** Frequency of positive cultures and clinical signs in vaginal and urine samples in Kerman and Shiraz province

		**Kerman**		**Shiraz**		**Total**	
		**No.**	**Percent**	**No.**	**Percent**	**No.**	**Percent**
Culture	Positive	16	16.5	8	15.1	24	16
	Negative	81	83.5	45	84.9	126	84
Sample type	Urine	15	15.5	0	0.0	15	10
	Positive	8	53.3	0	0.0	8	5.3
	Negative	7	46.6	0	0.0	7	4.6
	Vaginal	82	84.5	53	100	135	90
	Positive	8	9.75	8	15.1	16	11.85
	Negative	74	90.24	45	84.9	119	88.1
Clinical status	Symptomatic	55	56.7	8	15.1	63	42
	Positive	14	25.4	8	100	22	35
	Negative	41	74.6	0	0	41	65
	Asymptomatic	42	43.2	45	84.9	87	58
	Positive	2	4.8	0	0	2	2.3
	Negative	40	95.2	45	100	85	97.7

**Fig. 1:**
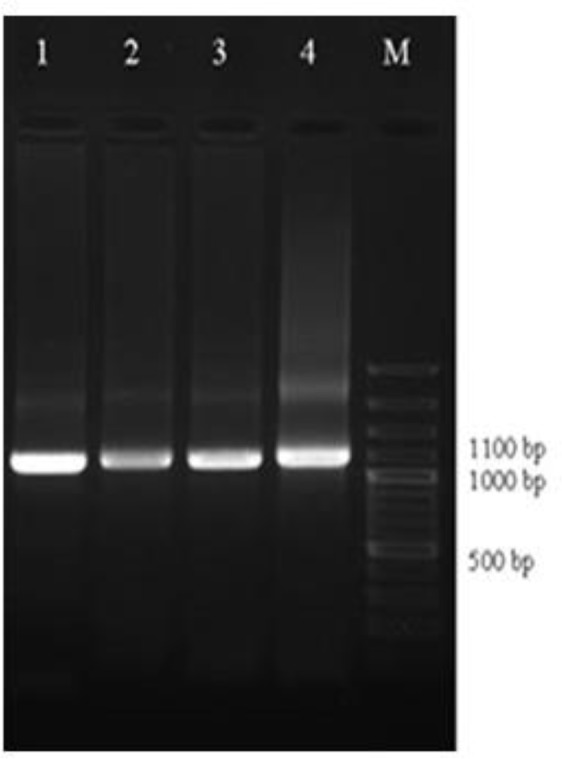
Agarose gel separation of representative nested PCR products of the actin gene from *T. vaginalis* isolates. Lane 1, positive control; lane 2–4, some *T. vaginalis* positive isolates from patients; M, 100 bp DNA ladder

All isolates were tested for the presence of *T. vaginalis* by culture and wet mount methods. Twenty-four (16%) isolates were positive for *T. vaginalis*; 16 (16.5%) and 8 (15.1%) isolates from Kerman and Shiraz city, respectively. All 24 microscopically positive urine and vaginal swab samples were successfully detected by nested-PCR (Fig. 1).

Among 63 symptomatic patients, 22 were positive for *T. vaginalis*, while 2 positive isolates were detected in asymptomatic patients. The findings revealed that there was a significant correlation (*P*-value=0.014) between clinical symptoms and positive cultures; so that having clinical symptoms increases the odds ratio of a positive culture of 6.63 (95% CI, 1.47–29.96).

### RFLP patterns

**Fig. 2:**
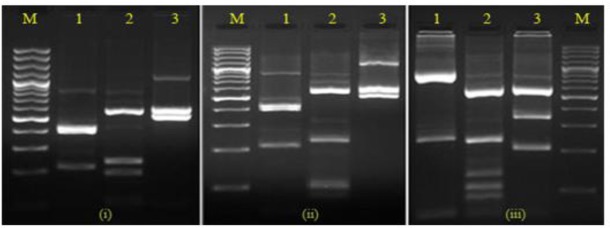
DNA fragment patterns of isolates after digestion of actin genotypes G(i), H(ii) and E(iii) on 3% agarose gel. M, 100 bp DNA ladder; lanes 1, 2 and 3, banding patterns after digestion with *HindII*, *RsaI* and *MseI* respectively

As shown in Fig. 2, digestion of the PCR product with *HindII* alone yielded three and/or four DNA fragments. All PCR products from clinical samples digested by the restriction enzyme *HindII* constantly represented a 60-bp and a 213-bp band. However, the restriction enzyme *RsaI* digested the PCR products into four, five or six fragments.

Again, fragments of 106-bp and 236-bp were representative of digestion with *RsaI* in all isolates. On the other hand, yielding two or three fragments were distinctive of cutting the PCR product by *MseI,* with a characteristic fragment of 581-bp. Overall, six different electrophoretic patterns were identified.

Genotype H was defined as a representative of pattern 2 after *HindII* and *RsaI* digestion and pattern 1 after *MseI* digestion. As such, genotype G was defined as representing pattern 2 after *HindII* and pattern 1 after *RsaI* and *MseI* digestion. The size of DNA fragments, pattern groups and actin genotypes of the *T. vaginalis* isolates are shown in Table 2.

**Table 2:** Size of fragments, pattern groups and actin genotypes of the *T. vaginalis* (extracted from Crucitti et al. 2008)

**Genotype**	**Restriction with *HindII* (bp)**	**Restriction with *RsaI* (bp)**	**Restriction with *MseI* (bp)**
A	827,213, 60	568,236,190, 106	581, 519
E	827,213, 60	568,236,106,103, 87	581,315, 204
G	426, 401,213, 60	568,236,190, 106	581, 519
H	426,401,213, 60	568,236,106,103, 87	581, 519
I	426,401,213, 60	452,236,190,116, 106	581, 519
M	426,401,213, 60	568,236,190, 106	581,333, 186
N	426,401,213, 60	568,236,106,103, 87	581,333, 186
P	426,401,213, 60	452, 236, 116, 106, 103, 87	581,333, 186

### T. vaginalis actin genotypes

The RFLP patterns of *T. vaginalis* specimens were designated for 16 isolates from Kerman, and for 8 isolates from Shiraz. Totally, six different genotypes were identified for the positive isolates. Table 3 shows the genotypes diversity among positive isolated based on their geographical distribution. Genotypes H (50%) and I (38%) were the most common genotypes identified from the two geographical locations studied. Moreover, mixed infections (50%) were only identified in only one of locations examined, in Shiraz.

**Table 3:** Frequency of the *T. vaginalis* actin genotypes in vaginal and urine samples in Kerman and Shiraz in symptomatic and asymptomatic patients

		**Symptomatic**	**Asymp*tomatic***	**Kerman**				**Shiraz**			
				**Urine**		**Vaginal**		**Vaginal**		**Total**	
		**N**	**N**	**N**	**%**	**N**	**%**	**N**	**%**	**N**	**%**
*T. vaginalis* genotypes	E	3	0	2	12.5	1	6.2	0	0.0	3	12.5
	G	2	0	0	0.0	2	12.5	0	0.0	2	8.3
	H	8	0	6	37.5	2	12.5	0	0.0	8	33.3
	I	3	2	0	0.0	2	12.5	3	37.5	5	20.8
	M	1	0	0	0.0	0	0.0	1	12.5	1	4.2
	N	1	0	0	0.0	1	6.2	0	0.0	1	4.2
	Mixed	4	0	0	0.0	0	0.0	4	50.0	4	16.7
	Total	22	2	8	50.0	8	50.0	8	100.0	24	100.0

### Phylogenetic diversity

**Fig. 3:**
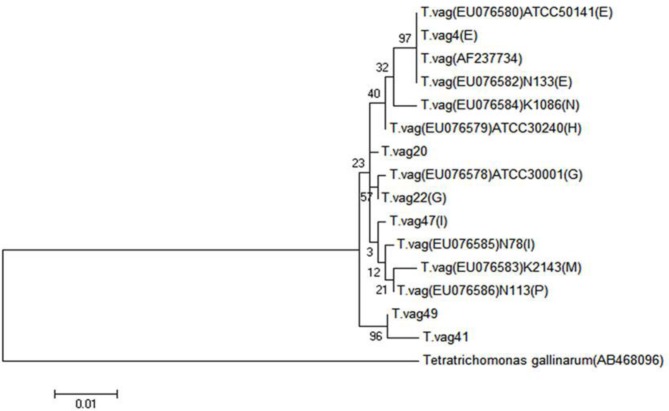
Phylogenetic tree of Kerman and Shiraz and reference trichomonad isolates, based on the actin gene. The six sequence types identified in this study are shown. Maximum-likelihood analysis was used to construct the tree. The scale bar indicates distance. Nodal support is given as a pp value

As evident from the phylogenetic tree (Fig. 3), although two isolates *T. vag* 41 and *T. vag* 49 were initially identified as M and N genotypes, respectively, in RFLP with 99% sequence homology with the reference strain K2143 (EU076583) and K1086 (EU076584) but were clustered in another clade in phylogeny (Fig. 3). The actin gene sequences of these two isolates are available in GenBank under the accession numbers: KC337042 and KC337O43.

## Discussion

Trichomoniasis is one of the most prevalent sexually transmitted diseases. Few studies have been conducted on virulence factors, transmission routes and sensitivity of different genotypes to metronidazole and presence of *T. vaginalis* virus, which is partly due to the lack of proper insight into the genetic mapping and genetic distribution of the parasite (19).

Although strain typing techniques are useful tools to study the epidemiology of parasitic infections, to our knowledge, a few molecular typing techniques for *T. vaginalis* including RAPD-PCR, PCR-RFLP, multilocus sequence typing (MLST) and sequencing polymorphism with a variety of markers such as ITS and microsatellite have been described (9–11, 20–24). PCR-RFLP technique based on the actin gene amplification provides a reproducible and sensitive method for strain typing of *T. vaginalis-*clinical isolates. In a previous report, digestion patterns of the actin gene with each restriction enzyme *Hind* II, *Rsa*I and *Mse*I, identified 8 different genotypes as the major genotypes in Zambia and Kinshasa, with G and E as the most prevalent genotypes, respectively (11).

In the current study, six different *T. vaginalis* genotypes were identified targeting the actin gene by PCR-RFLP technique. Different fragment patterns obtained from RFLP showed various types such as H, G, E, I, M, N confirmed by the designation of their nucleotide sequences (Fig. 2). Various distributions of *T. vaginalis* genotypes were found in isolates from Kerman and Shiraz, and the most frequent types were H and I genotypes, respectively. Genotypes A and P were not present in the targeted population studied while it was found in previous studies (11). The patterns of mixed genotypes were identified only in Shiraz but no evidence of mixed subtypes was observed in Kerman. However, mixed types and limited numbers of genotypes found in Shiraz may be related to probable occurrence of sex behavior in drug addicted or HIV subjects in that region.

An interesting feature of *T. vaginalis* is its high-level diversity. This heterogenetic protozoan has a specific population structure including two different types reported worldwide (19).

A considerable genetic diversity among isolates of six different genotypes was observed that might be due to the targeted population assessed. Such a discriminative ability of the current study is comparable to those, with ten different types and with eight types. *T. vaginalis* isolates using Single Stranded Conformation Polymorphism-PCR (SSCP-PCR) with ITS region showed two different patterns (13); in addition, the SSCP-PCR implemented with the actin gene. “According to the SSCP banding patterns and nucleotide sequencing, seven sequence types were detected among the isolates. Polymorphic nucleotide sites caused silent mutations and no amino acid substitution was observed” (25).

Overall, our study showed that in spite of the existence of a high level of genetic diversity in the *T. vaginalis* population, the genotypes were derived from two distinct clades; this is in agreement with other researchers (10, 20, 26).

Genotype I was the only genetic subtype identified from asymptomatic patients in Kerman with a significant difference (*P*<0.05).

Presence of different genotypes of *T. vaginalis* in Kerman with more symptomatic groups may be due to the heterogenicity of the population studied, which were from prisoners women with different geographic background. Additionally, sequence comparison of clinical isolate *T.vag* 4 showed 100% homology with the nucleotide sequence of the ATCC reference strain 50141(accession number EU076580); both were E-type.

Two isolates *T. vag* 41 and *T. vag* 49 were initially identified as M and N genotypes, respectively, in RFLP with 99% sequence homology with the reference strain K2143 (EU076583) and K1086 (EU076584) and nucleotide changes were about 10 and 7 bases from reference strain. However, since these two genotypes were clustered in another clade in phylogeny, it may indicate the presence of two unidentified novel genotypes in the population studied (Fig. 3).

## Conclusion

PCR-RFLP technique has an appropriate discriminative ability to detect different strains in populations and H type is the predominant genotype in the studied group. This study is confirming extensive diversity in the *T. vaginalis* and existence of two type’s genetic populations in the protozoa in Iran. Finally, this is the first report of a genomic analysis in Iranian isolates of *T. vaginalis* from symptomatic and asymptomatic patients using actin marker with RFLP technique in Kerman and Shiraz. Further studies are needed to interpret the association of a given genotype with regard to the outcome of clinical infections.
